# Trade-off between morphological convergence and opportunistic diet behavior in fish hybrid zone

**DOI:** 10.1186/1742-9994-6-26

**Published:** 2009-10-27

**Authors:** Emmanuel Corse, Caroline Costedoat, Nicolas Pech, Rémi Chappaz, Jonathan Grey, André Gilles

**Affiliations:** 1 Aix-Marseille Université, CNRS, UMR 6116 - IMEP, Equipe Evolution Génome Environnement, Centre Saint Charles, case 36, 3 place Victor Hugo, 13331 Marseille, France; 2 School of Biological and Chemical Sciences, Queen Mary, University of London, Mile End Road, London E1 4NS, UK

## Abstract

**Background:**

The invasive *Chondrostoma nasus nasus *has colonized part of the distribution area of the protected endemic species *Chondrostoma toxostoma toxostoma*. This hybrid zone is a complex system where multiple effects such as inter-species competition, bi-directional introgression, strong environmental pressure and so on are combined. Why do sympatric *Chondrostoma *fish present a unidirectional change in body shape? Is this the result of inter-species interactions and/or a response to environmental effects or the result of trade-offs? Studies focusing on the understanding of a trade-off between multiple parameters are still rare. Although this has previously been done for Cichlid species flock and for Darwin finches, where mouth or beak morphology were coupled to diet and genetic identification, no similar studies have been done for a fish hybrid zone in a river. We tested the correlation between morphology (body and mouth morphology), diet (stable carbon and nitrogen isotopes) and genomic combinations in different allopatric and sympatric populations for a global data set of 1330 specimens. To separate the species interaction effect from the environmental effect in sympatry, we distinguished two data sets: the first one was obtained from a highly regulated part of the river and the second was obtained from specimens coming from the less regulated part.

**Results:**

The distribution of the hybrid combinations was different in the two part of the sympatric zone, whereas all the specimens presented similar overall changes in body shape and in mouth morphology. Sympatric specimens were also characterized by a larger diet behavior variance than reference populations, characteristic of an opportunistic diet. No correlation was established between the body shape (or mouth deformation) and the stable isotope signature.

**Conclusion:**

The Durance River is an untamed Mediterranean river despite the presence of numerous dams that split the river from upstream to downstream. The sympatric effect on morphology and the large diet behavior range can be explained by a tendency toward an opportunistic behavior of the sympatric specimens. Indeed, the similar response of the two species and their hybrids implied an adaptation that could be defined as an alternative trade-off that underline the importance of epigenetics mechanisms for potential success in a novel environment.

## Background

Biologists have long recognized that introduced species may have major effects on native communities. If an invading species occupies the same niche as a native species, strong interactions are likely to occur [1]. Species are said to compete when they have negative effects on each other by consuming or controlling access to a limited resource. Low resource availability and a large overlap in fundamental niches make competition more likely. In these conditions we can expect two main outcomes as the result of contact between close competitors. First, one species may drive the other to localized extinction, as predicted by the competitive exclusion principle, which states that no two species can occupy the same niche indefinitely when resources are limited. Second, competition may lead to ecological character displacement [2,3], in which competition between similar individuals imposes disruptive selection on resource use and associated phenotypic characters, leading to divergence and reducing competition [4]. Character displacement allows close competitors to coexist by promoting divergence in resource use [5]. Phenotypic and life-history plasticity are generally important for successful animal invasions and freshwater fish commonly display high levels of plasticity [6,7].

*Chondrostoma t. toxostoma *is a threatened, protected endemic cyprinid species from southern France. Part of its distribution range was colonized at the end of the XIX's century by the invasive *Chondrostoma n. nasus*, from Eastern Europe. The mitochondrial divergence between the two species calculated from *cytochrome b *gene was about 7 My [8,9]. Where the two species are found in sympatry, a bi-directional hybridization phenomenon has been described in the Durance river (Rhone basin) [8]. The Durance river has been strongly structured by progressive urbanization and an increase in human activities along the river in the last 50 years. Various dams were constructed preventing fish from swimming upstream or downstream and leading to the formation of different water regulated environments. This situation promotes the coexistence of the two species in a "closed" environment (between dams) and makes both competition and hybridization phenomenon likely. Recently, Costedoat *et al*. [10] demonstrated the presence of the two parental species and different hybrid combinations in the Durance hybrid zone. Furthermore, they described a unidirectional change in the *Chondrostoma*'s body shape, regardless of the genomic combination (i.e. pure or hybrid), when compared to allopatric populations. Considering that the two species and their hybrid presented this body shape deformation, the authors hypothesized that it could be a consequence of environmental adaptation rather than hybridization. However, the authors did not analyze either the species distribution, or the body shape deformation in regard to the environmental pressure degree that could be defined in the Durance river. Indeed, it is possible to define a highly regulated (HR) part (downstream) which has numerous dams and weirs leading to a confined environment, with irregular alternation of water flow, where the two species strongly overlap (through space and time) and a less regulated (LR) part in which the water flow is not severely regulated and in which fish can notably migrate to some tributaries, (further upstream). The habitat of the two species is known to be different in the literature [11], with moderate to fast-flowing, large to medium-sized rivers with a rock or gravel bottom for *C. n. nasus *(*Cnn*) and fast and clear water rivers with a cobble bottom for *C. t. toxostoma *(*Ctt*). This situation raises two majors questions:

A century after the *Cnn *colonization in the Durance river what is the distribution of the two species and their hybrids in these two level of "anthropized" context (the LR and the HR zones)? Secondly, is there a difference in body shape between the specimen sampled in allopatry and those sampled in the two different anthropized parts of the river?

However, in the light of the differential environmental pressure in the Durance river (due to the fragmentation of the habitat), it appears obvious that others important parameters could interact with body shape. Indeed, morphology is also known to play a major role in determining the diet of a species, imposing physical constraints on the minimum and maximum size of prey that can be ingested, and affecting the efficiency of prey capture and consumption. A deep body shape is known to favor the consumption of benthic prey by increasing the manoeuvrability of the body, whereas a shallow body shape is likely to be beneficial for foraging on plankton [12,13]. Other important morphological characteristics define resource use: the mouth morphology, defined by the shape of the mouth [14-18] and the gape (maximal mouth dimensions) [19-21]. The mouth of the *Chondrotostoma *species is furthermore a diagnosed character. *Cnn *is defined by a straight inferior mouth and a lower lip with a thick keratinized sheath [11], often linked to a specialized diet mainly based on benthic diatoms [22], whereas the inferior mouth of *Ctt*, is arched (the lower jaw is often considered to be horseshoe-shaped -- first description Vallot [23]) and *Ctt *is known to feed on invertebrates and algae [11]. All these previous information underline the importance to obtain a good estimation of the "chondrostome diet behavior". However, dietary studies are difficult to carry out without direct observation of feeding behavior or without killing the animals. The fish of the Cyprinidae family have pharyngeal teeth, which crush everything ingested, making it difficult to recognize dietary components in the gut. For *Chondrostoma *species, we are also confronted with problems relating to species conservation status. The stable isotope analysis method presents advantages over conventional techniques in that it is compatible with non-lethal sampling and provides a short- or long-term integral picture (depending on the tissue used) of diet and metabolism [24-26].

Considering the Durance river context previously defined by differential anthropization levels, it seems relevant to test the modification and the interactions of the mouth morphology and diet behavior between the specimen sampled in allopatry and those sampled in the two different anthropized part of the river (the LR and the HR zones). To answer these questions, we performed a morphometric study on body and mouth shape (for which a new protocol was proposed) and used coefficient of condition and stable isotope analysis to determine the diet behavior considering the age and sex of each specimen. Modification of the different variable parameters and their interactions were tested for the *Chondrostoma *specimens present in the LR and the HR zones (representing the sympatric area) and those coming from the allopatric area.

## Results

### Specimen identification and population description

**Figure 1:**
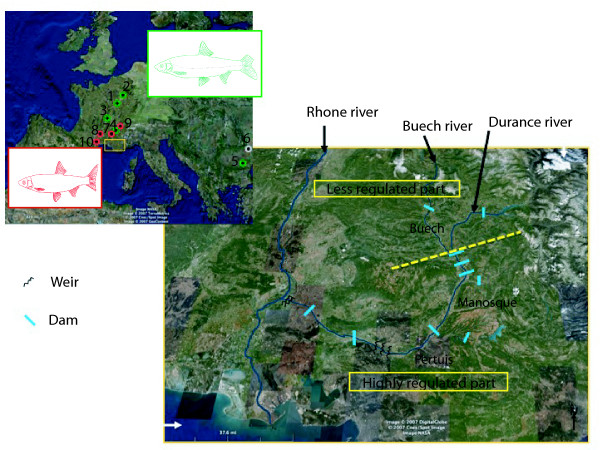
**Geographic distribution range**. Green color represents allopatric *C. nasus nasus *populations 1 = Chee; 2 = Flet; 3 = Allier; 4 = Usses; 5 = Tisza; Grey color represents *C. n. borysthenicum *populations 6 = Dniester (see text for more details). Red color represents allopatric *C. toxostoma toxostoma *populations 7 = Ain; 8 = Tarn; 9 = Doubs; 10 = Berre. Yellow square represents the hybrid zone (Durance river) with the split into Less Regulated part (Buech) and Highly Regulated part (Manosque-Pertuis).

The mtDNA sequences of specimens sampled in allopatry confirmed their identification (*Cnn *or *Ctt*), with the exception of the Dniester population (figure 1). The *cytochrome b *sequences obtained, confirmed that this population corresponded not to *C. nasus nasus*, but to its sister group (See Additional file 1: "Phylogenetic relationships between the Moldavian Dniester population samples and other *C. nasus *specimens"). We, therefore, used this population as an outgroup for the isotope and morphological analyses. The Genbank accession numbers of these Dniester population sequences are from GU112516 to GU112525.

Chi-squared tests did not show that the sex ratio is significantly different from 1 in the populations (χ^2 ^= 12.21, df = 7, P > 0.05; except for Chee and Tisza were there is an excess of males; χ^2 ^= 22.54, df = 9, P = 0.0085). Fish do not exhibit significantly different age distribution in the sympatric area (χ^2 ^= 32.19, df = 15, P = 0.080), with most individuals being between two and three years old. Three of the stations in allopatric conditions had older fish: Ain, Dniester and Tisza; and one had younger fish: Chee (χ^2 ^= 200.59, df = 33, P < 10^-6^).

### Diversity index of the two species and their hybrids in the Durance

The Shannon diversity index was significantly higher (P = 0.009) in the highly regulated (HR) part of the river (H_Ma-Pe _= 3.35) than in the less regulated (LR) part (H_Bu _= 2.93), meaning that there are more different hybrid classes represented in Ma-Pe stations (55 hybrid classes) than in the Buech (44 hybrid classes), the two parental species (*Cnn *and *Ctt*) being present everywhere.

### Body shape deformation

**Figure 2:**
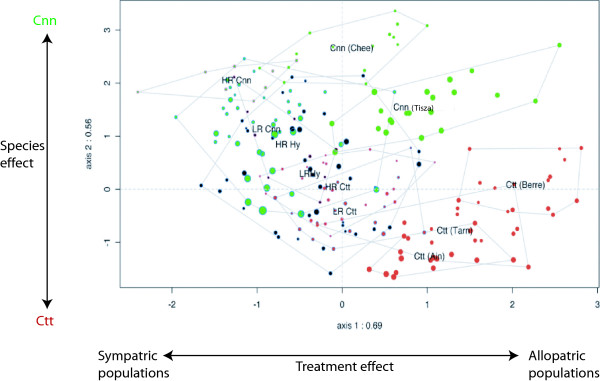
**Discriminant analysis for plastic body morphological characters**. Allopatric and sympatric populations including hybrids. Circle interior color: red = *Ctt*; green = *Cnn*; black: hybrids. Circle outline color: blue: LR part; pink: HR part. Circle diameter is proportional to the specimen size.

**Figure 3:**
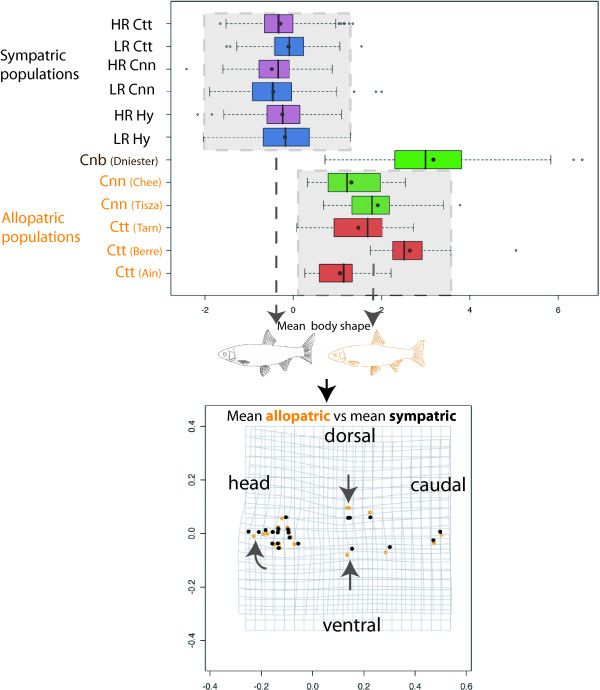
**Box-plot of body shape in function of groups**. Body shape is defined by axis of the linear discriminant analysis on treatment effect. Considered groups are: *Cnn *= *Chondrostoma nasus nasus*; *Ctt *= *Chondrostoma toxostoma toxostoma*; Hy = Hybrids; HR = Highly regulated river part; LR = Less regulated river part. Deformation grid: orange: allopatric mean specimen; black sympatric mean specimen. The shape differences have been exaggerated three-fold for better visualization. Arrows indicate the main deformations (tendency to a spindle-shaped body and the snout steered upward in the hybrid zone).

We carried out linear discriminant analysis on the populations considering all the allopatric and the hybrid zone populations. The first two axes were related to a species effect (*Cnn *vs *Ctt*) and to treatment effect (allopatric vs sympatric). We observed the tendency towards a change in body shape in the sympatric population compared to allopatric population as described in a previous study [10]. Indeed, all hybrid zone specimens displayed a similar deformation of body shape, tending to blur the species effect (figure 2). Linear discriminant analysis of the treatment effect allowed us to characterize the body shape deformation. This deformation (with a tendency to a spindle-shaped body and the snout steered upward) depended on the sampling stations and was more accentuated in the HR part than in the LR part, (mean value of -0.3306 for HR population and mean value of -0.1838 for LR ones; F_1,1106 _= 26.68; P < 10^-6^), figure 3.

### Mouth morphology

#### Mouth shape

**Figure 4:**
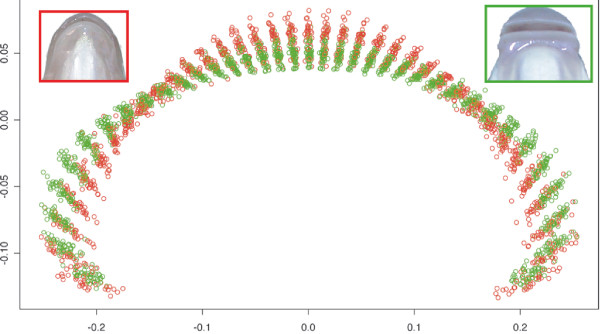
**Mouth morphology analysis**. The range of possible mouth shapes for the two species of *Chondrostoma*, corresponding to the superimposition of mouth landmarks after the procust superimposition of all allopatric specimens. Red circle = allopatric *Ctt*; green circle = allopatric *Cnn*.

**Figure 5:**
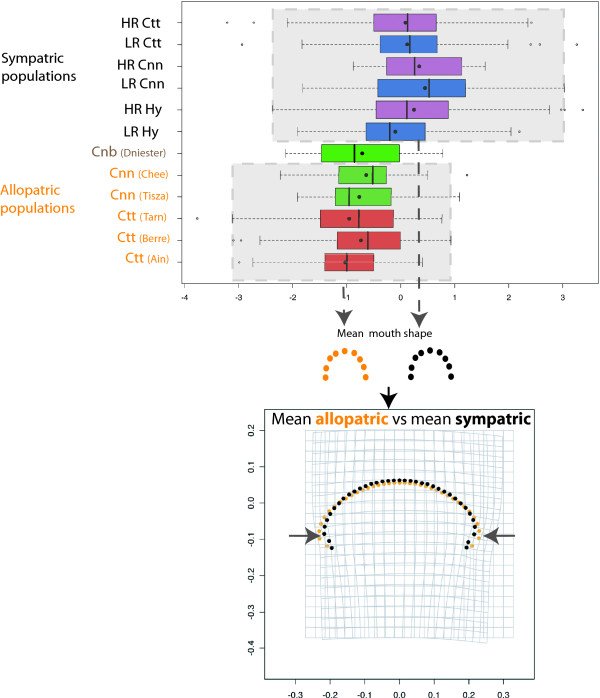
**Box-plot of mouth shape in function of groups**. Mouth shape is defined by the axis of the linear discriminant analysis on treatment effect. Considered groups are *Cnn *= *Chondrostoma nasus nasus*; *Ctt *= *Chondrostoma toxostoma toxostoma*; Hy = Hybrids; HR = Highly regulated river part; LR = Less regulated river part. Deformation grid: orange: allopatric mean mouth; black sympatric mean mouth. The shape differences have been exaggerated three-fold for better visualization. Arrows indicate the main deformations (corner narrow shape for hybrid zone specimens).

A superposition of the mouth shapes of all reference specimens is presented in figure 4 and represents the variation in mouth morphology observed in the two allopatric species. Despite an intra-species variance, we can discriminate the two species based on their mouth shape. Rather than a species effect in the hybrid zone we wanted to compare the treatment effect (i.e. allopatry vs sympatry) and determine if the body shape deformation in the hybrid zone was correlated to a mouth shape deformation. A difference in mouth morphology was observed between allopatric and sympatric specimens and corresponded to corner narrow shape (F_1,909 _= 122.53, P < 10^-6^, figure 5). This deformation is not species dependent (F_1,117 _= 1.45, P = 0.23, a detailed analysis of mouth shape deformation in function of hybrid combinations will be presented in Corse *et al*. in prep). This deformation of mouth morphology (R2 = 11.88), was less linked to treatment effect than the modifications observed in body shape (R2 = 53.39 for the body shape) and has been visualized figure 5. No significant differences were observed in the mouth morphology between specimens sampled in the HR part and those sampled in the LR part (F_1,735 _= 0.44; P = 0.51).

#### Mouth gape size

The gape of the mouth (ratio gape size on body size) presented a significant effect according to the groups (the two species and their hybrids; F_2,731 _= 45.68; P < 10^-6^) and was also significantly larger for specimens sampled in the LR part of the river than those sampled in the HR part (F_1,731 _= 4.53; P = 0.034), Additional file 2.

### Diet behavior

#### Coefficient of condition: K

In reference populations, with a correction for age effect, the mean K values for *Cnn *and *Ctt *were significantly different (respectively K = 12.50 and K = 9.84; F_1,136 _= 49.92 P < 10^-6^). In the hybrid zone, the K values were not significantly different and a species effect was not observed (K_*Cnn *_= 11.25; K_*Ctt *_= 11.27 and K_Hy _= 11.21; F_2,1095 _= 0.77; P = 0.4645). Significant differences were observed between the LR zone and the HR zone (respectively, K_LR _= 10.97. and K_HR _= 11.41; F_1,1095 _= 28.73 P < 10^-6^).

#### Stable isotopes: dC^13 ^and dN^15^

The reproducibility was high for the different aliquots, with generally less than 0.09‰ variability for d13C and 0.2‰ for d15N.

**Figure 6:**
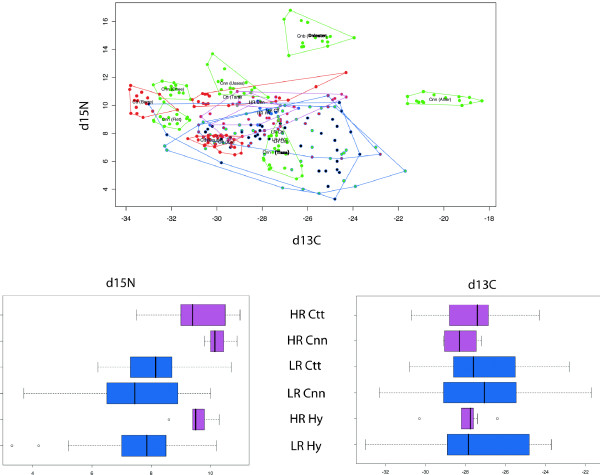
**Stable isotope analyses**. δ13C- δ15N bi-plots with individuals plotted based on their stable isotope signatures. Allopatric and sympatric populations. Circle interior color: red: *Ctt*; green: *Cnn*; black: hybrids; Circle outline color: blue: HR river part; pink: LR river part.

We generated δ13C- δ15N bi-plots, with individuals plotted on the basis of their stable isotope signatures (figure 6). The allopatric populations of the two species covered a very large range, both on the x-axis (δ13C axis from -33.84‰ to 18.32‰) and on the y-axis (δ15Í axis, from 6.47‰ to 16.81‰). Surprisingly, we were unable to discriminate between the two species due to a large overlap between populations, but we observed a strong population effect (43 of the 45 pairwise comparisons corresponded to a significant mean difference). We were able to differentiate between all the populations if we considered the two-dimensional space defined by the δ13C and δ15N axes (with the exception of the Doubs and Ain *Ctt *populations). This spreading over the isotope axes (greater on the δ13C axis than on the δ15N axis) did not reflect common hydrogeographic catchment areas, and no grouping of populations based on geographic links was observed.

The sympatric populations covered a large part of the total range delimited by the species in allopatry (figure 6). The variance was greater in sympatry than in allopatry (Additional file 3). Furthermore, for *Cnn *and hybrids the variance in the LR zone was higher than the variance in the HR zone, whereas no difference was observed for the *Ctt *specimens. Based on the δ15N, no significant difference was observed between the groups (δ15N_*Cnn *_= 8.069; δ15N_*Ctt *_= 8.335; δ15N_Hy _= 8.19; F_2,125 _= 0.33, P = 0.7171) but the difference was significant between the populations of the two parts of the hybrid zone (δ15N_HR _= 9.47; δ15N_LR _= 7.673; F_1,125 _= 22.36, P < 10^-6^). No significant difference was observed on δ13C for either of the groups (δ13C_*Cnn *_= -27.39; δ13C_*Ctt *_= -27.53; δ13C_Hy _= -27.56; F_2,125 _= 0.33, P = 0.72), nor for the different parts of the hybrid zone (δ13C_HR _= -27.69; δ13C_LR _= -27.42; F_1,125 _= 0.04, P = 0.84).

### Variable interactions

We found a significant correlation (r = -0.36; P < 10^-6^) between the treatment effect on body shape and the treatment effect on the mouth shape of the specimens (Additional file 4). However, no correlation was found between the treatment effect on body shape and the stables isotopes (neither for the δ15C nor for the δ13N) no matter from where the specimens were sampled. No correlation was observed between the size of the gape and the stable isotope analysis, and no direct link could be made between diet behavior (visualized with the stable isotope analysis) and the relative "robustness" or degree of well-being (represented by K) of the fish (data not shown).

## Discussion

### Competitive exclusion principle vs ecological character displacement

The habitat of the two Chondrostomes is known to be sufficiently different [11] to expect the endemic *Ctt *to be predominantly in the LR part of the river (upstream) and the invasive *Cnn *into the HR part of the river (further downstream). However, we did not observe this distribution but the total opposite. Despite the presence of the two species throughout the river, *Ctt *was mainly present in the HR part (72% of *Ctt*; 4% of *Cnn *and 24% of hybrids) whereas in the LR part the two species were more equally represented (33% of *Ctt*; 36% of *Cnn *and 31% of hybrids), calculated from [10]. The overwhelming majority of *Ctt *in the HR part could indicate the beginning of a competitive exclusion process in this more disturbed environment, with the curious advantage of the endemic species. The Shannon diversity index calculated in this study presents a stronger diversity in the HR part than in the LR part, indicating more different hybrid classes downstream than upstream (even if there is no significant difference between the two parts of the river in terms of rate of hybrids, calculated from [10]). These results illustrated that the different sympatric zone environments could have an influence on the distribution of the species and that environment pressure, associated with competition and apparition of new inter-species combinations (via hybridization), led species outside of their ecological preferendum (ecological transgressive segregation).

Furthermore, regardless of the species, we observed a body shape deformation between the two treatments (allopatric vs sympatric), with a tendency to a spindle-shaped body with a snout steered upward in the hybrid zone. This deformation was environmentally dependent with a stronger accentuation in the HR part than in the LR part, but concerned both species and their hybrids. This over-all body shape convergence is not in accordance with the ecological character displacement theory wherein when two species overlap geographically the difference between them is accentuated in the zone of sympatry and weakened, or lost entirely, in the parts of their ranges outside this zone [2-4]. We can not assume that this result is due (or exclusively linked) to the hybridization process because the intensity and directionality of hybridization is not the same throughout the sympatric zone [10]. Adaptation for morphological specialization (here a spindle shaped body with a snout steered upward) have been explained in a wide range of fish species by a process of competition between co-existing populations which leads to resource partitioning [27]. However, we can also exclude the effect of species competition, because we did not observe a phenotypic divergence that could lead to reducing competition (e.g. benthic vs limnetic shape as found in sticklebacks [28]). But on the contrary all the specimens presented the same morphological tendency. Environmental pressure seemed to have more influence on the phenotype of the species complex than hybridization and competition. However, the slight difference in morphology observed between specimens sampled in the LR part of the river and those sampled in the HR part, tended to illustrate that the "regulated" effect of the river is not the main cause of this new morphology.

### Specialization in allopatric zones

To improve interpretation of the sympatric results we detailed the diet behaviour of the two species based on the different allopatric populations. We did not observe a specific stable isotope signature and the direct link one species/one diet behaviour could not be assumed with our results. Some studies illustrated cases in which significant difference between the diets of two species was not observed based on muscle but was detected on liver (Cf. notably in [24]). However, even if we did not observe a species effect, we observed a population effect that led us to think that the used of muscle instead of other tissue is not the most parsimonious hypothesis to explain this pattern. If we assume that the baseline does not differ significantly between different tributaries or rivers, our results indicate different diets for each of the allopatric populations. If we assume a significant difference in the baseline between tributaries and rivers, we cannot compare trophic levels between populations. However, what is crucial in both cases, is that the dietary behaviour of the various allopatric populations is markedly similar for the specimen belonging to the same population and characterized by a narrow range (tendency to a specific diet behaviour) regardless of the considered species. In summary, the invasive and the endemic species displayed similar global plasticity when all populations were considered. The general larger variances observed in the sympatric populations compared to allopatric populations could indicate that the specimens presented a more opportunistic dietary behaviour. These variances could be the result of hybridization, competition between species (sympatry), the water regulated effect, or a combination of these three factors.

### The trade-off in fish hybrid zone: morphological convergence versus opportunistic diet behaviour

The tendency toward a more "limnetic" morph in the hybrid zone is, moreover, linked to a mouth shape deformation. Thus, we hypothesized that the phenotype deformation could depend on a significant alteration of food availability in the river and, therefore, on diet behaviour. The most obvious impact of introduced fish on native species is through competition for food [29]. Stable isotope analyses made it possible to determine both trophic levels (nitrogen ratio) and the spatial origin of food (carbon ratio) [30-34]. Furthermore, as we tested the age and sex difference for all the populations (none are significant), the difference observed in term of morphology, coefficient of condition, or stable isotope could not be due to these characteristics but to genetic and/or environmental effects. Comparisons of the dietary behaviour of the *Chondrostoma *"complex" in the sympatric zone did not lead to the identification of a species effect, but we observed a wide overlap of isotope signature between *Cnn*, *Ctt *and hybrid specimens. This result was confirmed by the coefficient of condition that presented no significant species effect in the sympatric zone.

In summary, we observed a convergence in body and mouth shape in the sympatric zone, as well as a large variance in diet behaviour. However, no correlation was found between the sympatric effect on body shape and the stable isotopes (neither for the δ15C nor for the δ13N) regardless of where the specimens were sampled. Moreover, this morphological convergence could not be explained by the resource use diversity, also known as "individual specialization", in which it is assumed that a population is composed of ecologically heterogeneous individuals [35,36]. The absence of relation between morphology convergence and opportunistic diet behaviour appeared to be more complex than expected in the sympatric zone (whatever the anthropic level) and even if the species effect tended to be blurred in the sympatric zone some characters kept this species information (e.g. the mouth gape).

### To move towards the epigenetic base of adaptation

Dietary shifts, from specialist to opportunist/generalist behaviour, are often seen as a sign of invasion aptitude [37,38], favouring the colonization of new areas [39,40] and the results we obtained here did not confirm this tendency. This surprising result may be accounted for by plasticity in dietary behaviour that has been described globally for other cyprinid species [41]. Elshoud-Oldenhave and Osse [42] were the first to show that many teleosts adjust their alimentary strategy according to the environment in which they find themselves and the prey it contains (and not in function of their invasive aptitude). Liem [43] described this multiple potential strategy as "modulatory multiplicity". The use of this strategy makes teleost species highly versatile and could explained why we observed this populations difference regardless of the considered species even if at the population scales the diet behaviour appeared to be very specialised.

Furthermore, all the results obtained tend to show that water flow is not directly responsible for the specimen plasticity observed in the hybrid zone as was previously hypothesized [10]. However, we could not exclude an indirect effect of this parameter due to the influence of alternate patterns of clogs and leaches, which characterize this river. The Durance River is defined as an untamed Mediterranean river, notably under the influence of floods and drought periods. The major bed of the Durance is very large and the minor bed often oscillates over it. Thus, in this random change of water bed, the resilience periods vary in length and fish are dependent on the abundance of algal and invertebrate communities. We can therefore assume that the tendency toward an opportunistic dietary behaviour could be an adaptation to the rapid and stochastic changes in the river's benthic communities.

Finally, the results reported in this study deal with the theoretical concept based on the study of epigenetic mechanisms. These have been reported to have the potential to create either the so-called maintenance phenotype if the environmental conditions are unstable, poor or unpredictable (a typical situation for each invader, and in the case of this study also for *Ctt *that is likely to feel uncomfortable with the new competitor *Cnn*), or alternatively, the so called dispersal phenotype equipped to deal with conditions of dispersal in rich, unexploited "luxury environments". This concept has been further developed for fish, as the ontogenetic point was taken into account. As a result, a theory of alternative ontogenies has been proposed to explain the capacity of fish to create either specialized or generalized phenotypes and/or life-histories (e.g. in [44]). Finally, this theoretical concept has recently been applied to explain the success of some invasive fish and illustrate how biological invasion can also affect the invader itself, resulting in both genetic and epigenetic changes (e.g. in [45]). Epigenetic mechanisms (that can lead to alternative ontogenies) may have important implications for potential success in novel environment. The bighead goby study [45] and this *Chondrostoma *study tended to demonstrate the importance of considering such mechanisms in species adaptation and more globally in biological invasion studies and conservation biology.

## Materials and methods

### Data collection, study area and individual identification

We studied 1330 chondrostomes sampled in allopatry and sympatry. A subset of these samples came from [10], the rest represent new populations. The detail of population and sample size for each marker studied is presented in figure 1 and table 1. All specimens used for isotope analysis were stored at -80°C except those from Dniester and Berre, which were stored in 90% ethanol.

**Table 1:** Sampled populations and sample size.

**Pop.**	**Treatment**	**N DNA**	**N Sex**	**N Age**	**N Body shape**	**N K**	**Study**	**N Mouth shape**	N CN*	**Study**
Ain *Ctt*	Allopatry	30	30	29	30	29	This study	22	15	This study
Berre *Ctt*		31	0	29	31	29	This study	28	15	
Chee *Cnn*		30	17	30	29	30	This study	30	15	
Tisza *Cnn*		24	19	24	24	23	This study	18	15	
Tarn *Ctt*		27	22	27	26	27	This study	21	15	
Dniester *Cnb*		29	0	12	26	12	This study	26	15	
LRHY	Sympatry	136	110	133	134	126	[10]	107	42	
HRHY		169	79	169	166	169	[10]	115	10	
LRCnn		146	96	141	145	135	[10]	109	40	
LRCtt		154	114	153	154	143	[10]	117	18	
HRCnn		34	14	33	34	33	[10]	22	8	
HRCtt		520	309	514	508	513	[10]	296	26	
**Total**		**1330**	**810**	**1294**	**1307**	**1269**		**911**	**234**	

*Cnn *= *Chondrostoma nasus nasus*; *Ctt *= *Chondrostoma toxostoma toxostoma*; Hy = Hybrids; HR = Highly regulated river part; LR = Less regulated river part. * For CN analysis we added one allopatric *Ctt *population (Doubs N=15) and three *Cnn *populations (Allier N = 15, Flet N = 15, Usses N = 15). See text for details of the identification of *C. n. borysthenicum *in Dniester river.

Whenever possible we determined the sex (defined after dissection or canulation method cf. [10]) and the age (defined by scalimetry) of the specimen. Morphology and diet behaviour could be influenced by these two factors, so we needed to check the sex ratio and the age range in each population before comparing them.

The identification of the chondrostomes of new allopatric populations was confirmed (i.e. to ensure that allopatric specimens were "pure") by DNA extraction, with amplification and sequencing of the mitochondrial *cytochrome b *gene (details of the protocols in [46]). Hybrid zone specimens (Durance River) were identified in Costedoat *et al*. [10] by combining the alleles obtained for five molecular markers (mitochondrial *cytochrome b *gene and four nuclear introns). The authors identified the two parental species and 65 hybrid classes in the Durance. These 65 detailed hybrid classes were used in this study to calculate the Shannon diversity index to compare the diversity in the LR and HR parts of the sympatric zone. However, for statistical accuracy in the study of morphology and diet behaviour, we pooled all hybrids together because there were too few specimens in some of the 65 categories.

### Morphological analysis

#### Body shape

Body shape was analyzed by the landmark-based geometric morphometric method. We defined 21 homologous landmarks on the body (cf. in [10]). All landmarks were digitized, using TpsDig software [47]. Landmark-based geometric morphometric methods were used to capture information about shape, by obtaining the x and y coordinates of homologous landmarks. Differences in the sets of coordinates between specimens due to scaling, rotation and translation were eliminated by a typical geometric morphometric approach [48,49] in which the specimens were placed in a procruste superimposition on the iteratively estimated mean reference form, using the generalized procruste analysis (GPA) procedure. Points representing landmark configurations were then projected into Euclidean tangent space approximating the curved shape space.

#### Mouth shape

We investigated whether the difference in mouth morphology between the two *Chondrostoma *species (more precisely the lower lip shape) was correlated to dietary behaviour. This required a quantification of the difference in shape. All previous comparisons of mouth morphology in the *Chondrostoma *genus were subjective (simple description of the morph, see [50]). We characterized differences in mouth shape objectively (as was done for the body shape), using a morphometric approach based on the landmarks method. The main problem encountered with this approach is the definition of homologous points along the line of the mouth. In our case, the two lip corners are good candidates for homology between the mouths of the two species. We overcame the problem of homologous points by placing tracing paper with a semi-radian drawn on it over the image of the mouth (we used Adobe Photoshop 5.0 for this superimposition). We then adjusted the diameter of the semi-radian to the width of the mouth, fitting the extremities of the radian to the two homologous points (lip corners). The semi-radian was divided by 37 lines (every 5°) and the intersection of each line with the edge of the lower lip was digitized with TpsDig version 1.40 [47]. This procedure was repeated for all the specimens studied, making comparisons of mouth shape possible.

### Diet behaviour analysis

#### Stable isotope analysis (SIA) method

The two main elements used in stable isotope analysis for ecological research (and particularly for studies of the diet of fish [24,30,31,51-54] are carbon and nitrogen. Isotope ratios are reported in delta notation as per international standards: carbon from the Pee Dee limestone formation [55] and atmospheric nitrogen [56]. Nitrogen ratio is considered to characterize trophic level, whereas carbon ratio is considered to reflect the spatial origin of food [30-32,51,53,57,58].

In this study, we used 294 chondrostomes for carbon and nitrogen stable isotope analysis. These fish were chosen because of the possibility to use muscle tissue [58] and correspond to specimen sampled in 2001. The fish captured after 2001 were analyzed on the fields and then released, we did not used them for stable isotope analyses (but they were used for morphological study). Freezing is the ideal method for preserving specimens, but storage in ethanol does not seem to affect stable isotope ratios [59]. The two populations stored in ethanol (Dniester and Berre) were therefore included in this analysis. Muscle tissue was collected from the dorsal musculature posterior to the dorsal fin. Muscles were dehydrated by drying in an oven at 60°C for two days and were then crushed with individual sterile breakers. Samples must be dried sufficiently for grinding into a fine powder. Dried samples were sent to the Colorado Plateau Stable Isotope Laboratory of Northern (Arizona University) for the determination of % N, % C, δ15N, and δ13C by isotope-ratio mass spectrometry.

First we analyzed the stable isotope data obtained for the allopatric populations, to estimate the isotope range (dietary behaviour) of each species. Then we integrated the results obtained for the sympatric zone, for which results were analyzed as a function of genotype (*Ctt*, *Cnn *or hybrid) and sampling station (highly regulated -HR- part or less regulated -LR- part).

### Coefficient of condition: K

The relative robustness, or degree of well-being, can be expressed as the "coefficient of condition" [60]. The formula most often used is: K = W/L3 (W = the weight in grams; L = the standard length in decimeters).

### Statistical analyses

Description of shape (body and mouth) variability was performed using linear discriminant analysis on group (*Cnn*, *Ctt*, Hy), "fragmentation"(HR river part and LR river part) and station variables. We compared the mean and variance for our variables of interest (isotopic signatures, shape deformation axes, mouth gape size, coefficient of condition) by way of analysis of variance. The corresponding factors were respectively defined by the species, the treatment and the fragmentation. If necessary, log transformations were performed to achieve homoscedasticity of the residuals. To take into account the incomplete design of the sampling scheme, effects were tested using a type III sum of squares. For each ANOVA, a global test was hence performed, followed, if necessary, by pairwise comparisons, (using Benjamini-Hochberg correction for multiple comparisons, [61]). Correlation between quantitative variables was analyzed using the Bravais-Pearson correlation coefficient. Shannon index was compared between groups by way of permutation test [62].

All statistical analyses were performed using R [63]. Deformation grids were visualized using the Tps grid function of the R shape package.

## Competing interests

The authors declare that they have no competing interests.

## Authors' contributions

EC and CC carried out the study and drafted the manuscript. AG Conceived the experiments. CC and AG designed the experiments and performed the manuscript preparation. EC, CC and NP analysed the data. EC, CC, JG and NP contributed to reagents/materials/analysis tools. EC, CC, NP, RC and AG performed the field work. RC participated to the financial support.

## Supplementary Material

Additional file 1 **Phylogenetic relationships between the Moldavian Dniester population samples and other *C. nasus *specimens**. We carried out pairwise sequence comparisons, using the neighbor-joining method, based on Kimura-2-parameter distance model on *cytochrome b *data set. We tested the topology of the tree produced, by a non-parametric bootstrap method (only values higher than 50 are represented). Click here for file

Additional file 2 **Box-plot of mouth gape in function of groups**. Mouth gape is defined by the axis of the linear discriminant analysis on treatment effect. Considered groups are *Cnn *= *Chondrostoma nasus nasus*; *Cnb*: *C. n. borysthenicum; Ctt *= *Chondrostoma toxostoma toxostoma*; Hy = Hybrids; HR = Highly regulated river part; LR = Less regulated river part. Click here for file

Additional file 3 **d13C and d15N variance by groups**. *Cnn *= *Chondrostoma nasus nasus*; *Ctt *= *Chondrostoma toxostoma toxostoma*; Hy = Hybrids; HR = Highly regulated river part; LR = Less regulated river part. Click here for file

Additional file 4 **Correlation between body shape and mouth shape's treatment effect**. Circle interior color: red = *Ctt*; green = *Cnn*; black: hybrids. Circle outline color: blue: LR part; pink: HR part. Circle diameter is proportional to the specimen size. Click here for file
