# Supplementary figures and images for: Trade-off between morphological convergence and opportunistic diet behavior in fish hybrid zone
Source: Front Zool. 2009 Oct 27;6:26. doi: 10.1186/1742-9994-6-26 (PMC2773775; doi:10.1186/1742-9994-6-26)

# Additional file 1

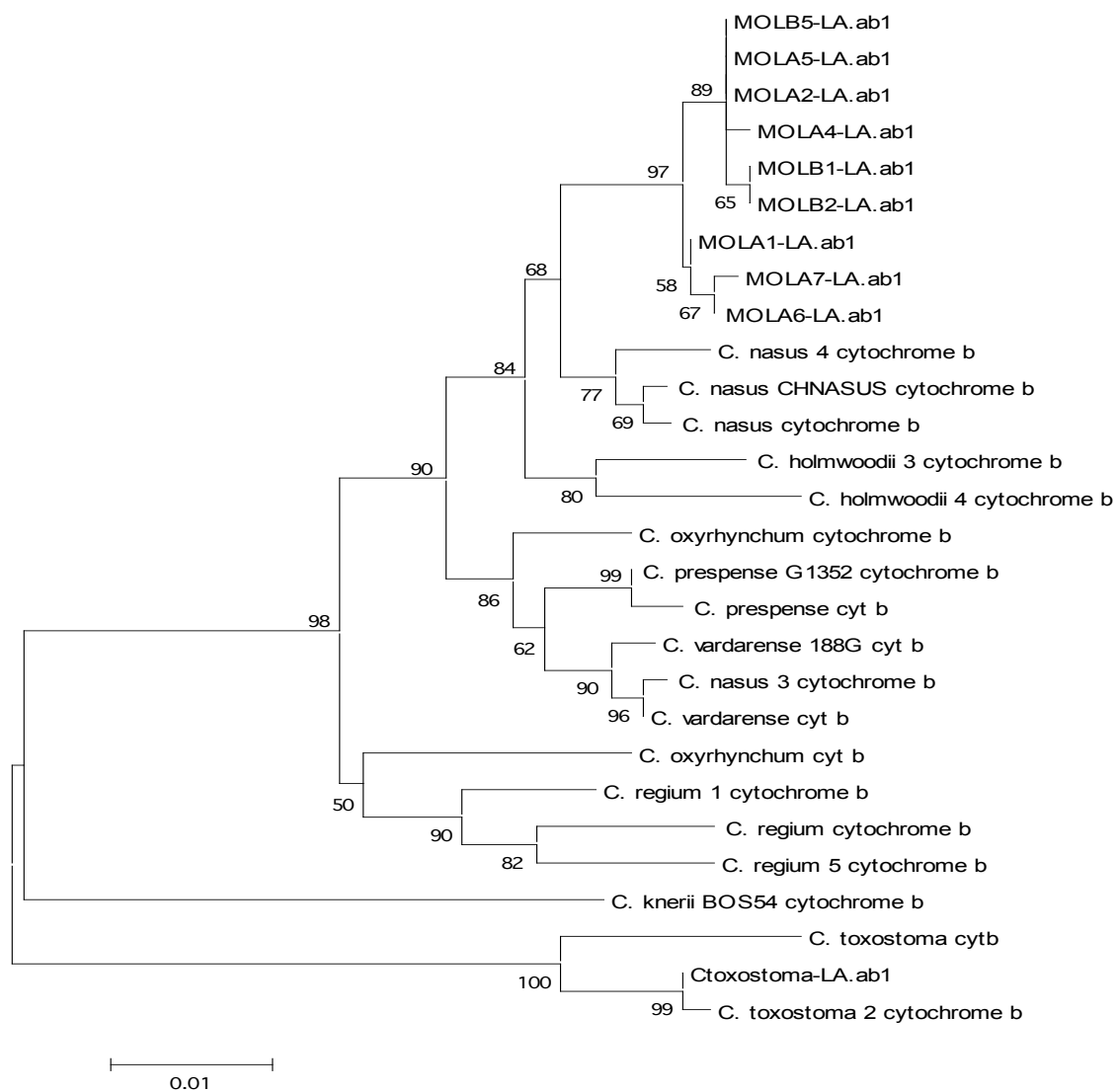

Supplement: Additional file 1 — Phylogenetic relationships between the Moldavian Dniester population samples and other C. nasus specimens. We carried out pairwise sequence comparisons, using the neighbor-joining method, based on Kimura-2-parameter distance model on cytochrome b data set. We tested the topology of the tree produced, by a non-parametric bootstrap method (only values higher than 50 are represented). [file 1742-9994-6-26-S1.PDF]

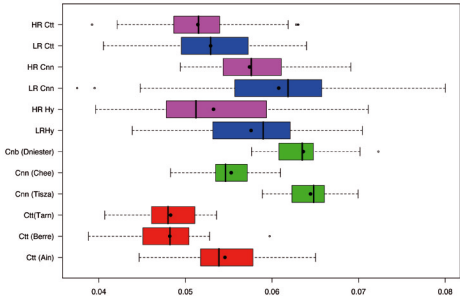

Supplement: Additional file 2 — Box-plot of mouth gape in function of groups. Mouth gape is defined by the axis of the linear discriminant analysis on treatment effect. Considered groups are Cnn = Chondrostoma nasus nasus; Cnb: C. n. borysthenicum; Ctt = Chondrostoma toxostoma toxostoma; Hy = Hybrids; HR = Highly regulated river part; LR = Less regulated river part. [file 1742-9994-6-26-S2.PDF]

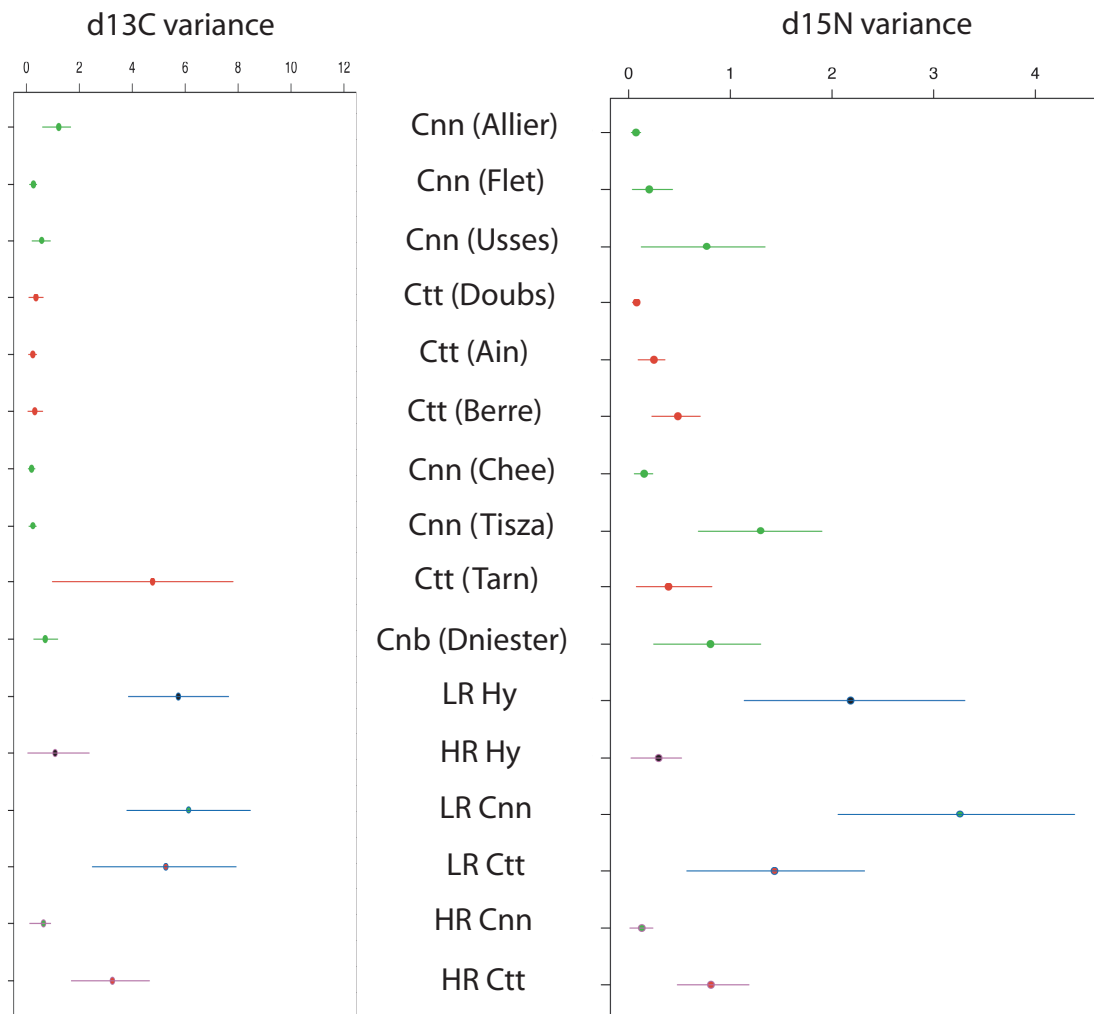

Supplement: Additional file 3 — d13C and d15N variance by groups. Cnn = Chondrostoma nasus nasus; Ctt = Chondrostoma toxostoma toxostoma; Hy = Hybrids; HR = Highly regulated river part; LR = Less regulated river part. [file 1742-9994-6-26-S3.PDF]

$r = -0.36$   $P = 0$

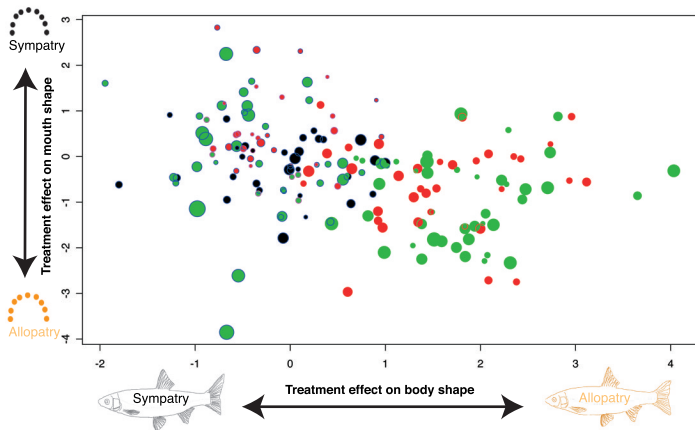

Supplement: Additional file 4 — Correlation between body shape and mouth shape's treatment effect. Circle interior color: red = Ctt; green = Cnn; black: hybrids. Circle outline color: blue: LR part; pink: HR part. Circle diameter is proportional to the specimen size. [file 1742-9994-6-26-S4.PDF]
